# Phylogeny-guided interaction mapping in seven eukaryotes

**DOI:** 10.1186/1471-2105-10-393

**Published:** 2009-11-30

**Authors:** Janusz Dutkowski, Jerzy Tiuryn

**Affiliations:** 1 Institute of Informatics, University of Warsaw, Banacha 2, 02-097 Warsaw, Poland

## Abstract

**Background:**

The assembly of reliable and complete protein-protein interaction (PPI) maps remains one of the significant challenges in systems biology. Computational methods which integrate and prioritize interaction data can greatly aid in approaching this goal.

**Results:**

We developed a Bayesian inference framework which uses phylogenetic relationships to guide the integration of PPI evidence across multiple datasets and species, providing more accurate predictions. We apply our framework to reconcile seven eukaryotic interactomes: *H. sapiens*, *M. musculus*, *R. norvegicus*, *D. melanogaster*, *C. elegans*, *S. cerevisiae *and *A. thaliana*. Comprehensive GO-based quality assessment indicates a 5% to 44% score increase in predicted interactomes compared to the input data. Further support is provided by gold-standard MIPS, CYC2008 and HPRD datasets. We demonstrate the ability to recover known PPIs in well-characterized yeast and human complexes (26S proteasome, endosome and exosome) and suggest possible new partners interacting with the putative SWI/SNF chromatin remodeling complex in *A. thaliana*.

**Conclusion:**

Our phylogeny-guided approach compares favorably to two standard methods for mapping PPIs across species. Detailed analysis of predictions in selected functional modules uncovers specific PPI profiles among homologous proteins, establishing interaction-based partitioning of protein families. Provided evidence also suggests that interactions within core complex subunits are in general more conserved and easier to transfer accurately to other organisms, than interactions between these subunits.

## Background

Protein-protein interactions are essential to most cellular processes. Thus large-scale PPI networks can greatly contribute to our understanding of the cellular machinery at systems level. Experimental techniques such as yeast two-hybrid assays [1-4] and TAP-MS [5,6] have generated large amounts of binary PPIs and protein complex data, providing the first snapshots of eukaryotic interactomes. Unfortunately, the available experimental techniques are far from perfect, both in terms of their accuracy, as well as coverage. For instance, the yeast interactome has recently been estimated to contain from around 37,000 up to even 75,500 protein interactions between approximately 6,000 proteins [7]. Although already over 80,000 yeast PPIs have been reported, given the estimated false positive rates of the experiments, the yeast interactome is suggested to be roughly 50% complete [7]. Using a more conservative definition and omitting indirect co-complex associations, the authors of [8] estimate the number of yeast interactions to be ~18,000 and conclude that three idependent Y2H assays cover only around 20% of this amount. In case of human, the entire interacome is estimated to be covered in roughly 10% [7,9]. Furthermore, many doubts and criticism have been expressed in the literature regarding the low overlap between independent screens - originally attributed to a high false-positive rate of these experiments [10-12]. More recent studies (e.g. [8]) suggest that the low overlap can largely be explained by low sampling sensitivity and differences in assay types. Considering all mentioned limitations, none of the existing experimental systems can provide a complete and error-proof interaction map of a complex organism within reasonable time and respecting budget limitations. As recently estimated, around 20 independent proteome-scale screens would be required to reliably identify each mappable interaction in a moderately-sized interactome of *Drosophila melanogaster *[13].

Simultaneously with the development of experimental techniques, computational methods for predicting PPIs have emerged [14-16]. These approaches complement experimental methods and can be used to validate noisy data, as well as to select new targets for screening experiments [15]. Available computational techniques exploit various sources of evidence. Among them are ones based on genomic data [17,18], protein sequences [19,20], phylogenetic profiles [21], and classification-based approaches [22-24]. Other methods explore the premise that interacting proteins often co-evolve and thus similarity of phylogenetic trees can be used to infer interactions [25-27]. Approaches using maximum likelihood estimation (MLE) for inferring the probability of domain-domain interactions have been presented. The first of such analysis was performed in [28], where the authors used yeast PPI data to estimate the probability of domain-domain interactions, and subsequently predict the interactions between proteins. Finally, multiple data sources have been integrated in a Bayesian framework in [29]. The last concept was further extended and applied to a wide range of heterogeneous data types from multiple species to construct comprehensive databases of functional associations [30,31].

In this study we are specifically interested in techniques which integrate and transfer PPI evidence across species. In its simplest form, this idea is implemented in the interolog (the term interlog is also used) mapping approach [32], which predicts an interaction between a pair of proteins (a, b) if in another species there exists a known interaction between a pair (a', b'), where a' and b' are orthologs of a and b, respectively. The transfer of PPI evidence across species can also be achieved at the level of conserved domains. In [33] the authors devised a maximum likelihood method, similar to [28], but using data from multiple organisms. In summary, the method estimates the probability of interactions between each pair of considered domains, based on the PPI evidence from multiple species. Inferred domain-domain interactions constitute integrated evidence, which is in turn used to predict protein-protein interactions. A similar method, but using heterogeneous data sources (including protein fusion and Gene Ontology annotations), was used in [34]. In general, combining interaction evidence from different species makes PPI predictions more robust to experimental noise. False positive observations are unlikely to be reproduced across multiple species [35]. Furthermore, evolutionarily conserved interactions are expectedly biologically significant. Evolutionary pressures are more likely to constrain functional units such as protein complexes rather than single interactions [36]. Hence, if an interaction has experimental support in datasets from diverse species, it is likely to be part of a significant functional module. Highly probable interactions identified in a subset of species can be transferred to other species [37], as was done in [38] to predict missing interactions within conserved protein modules.

We present an approach which uses protein family phylogenies to accurately map PPI evidence between homologous proteins. Contrary to previous studies [25-27], the phylogenies are not used to assess protein co-evolution, but to account for evolutionary relationships when integrating data from different organisms. Our current work builds on previously proposed CAPPI framework for comparing PPI networks across species [39]. CAPPI is based on a duplication and divergence model which mimics the processes by which most protein interactions are formed i.e. by copying from ancestral interactions during protein duplication and subsequently being sustained or lost over time. Using this model we can naturally incorporate inter-dependencies between PPIs and study the available data in evolutionary context. The only previous works based on these principles are [39] and [40] both of which concentrated on inferring ancestral states of the protein interaction network (the analysis in [40] was limited to a single protein family). Our current work presents the first application of the duplication and divergence model towards genome-scale inference of PPIs in extant species.

We use our framework to integrate and infer new PPIs in seven eukaryotes: *Homo sapiens*, *Mus musculus*, *Rattus norvegicus*, *Drosophila melanogaster*, *Caenorhabditis elegans*, *Saccharomyces cerevisiae *and *Arabidopsis thaliana*. We perform a comprehensive validation of our predictions using a GO-based functional similarity measure and assessment based on reference datasets of binary and co-complex PPIs. The obtained results demonstrate CAPPI's ability to identify a large percentage of known interactions in a blind test and provide new hypothesis for experimental verification when all known data is integrated. Our method shows a significant advantage over the standard interlog mapping approach and a maximum-likelihood domain-oriented method. We also analyze specific examples of valid PPI predictions in well-characterized complexes in yeast and human (proteasome, endosome and exosome), and show that core subcomplexes can be accurately recovered based solely on the data from the other species (i.e. without any use of the experimental data from the species of interest). Many of the between-module interactions (possibly species-specific) are harder to transfer from distant organisms. Finally, based on our predictions, we present hypothesis on new proteins interacting with the putative SWI/SNF chromatin remodeling complex in *A. thaliana*. Our results are freely available at http://bioputer.mimuw.edu.pl/cappi.

## Results and Discussion

**Figure 1:**
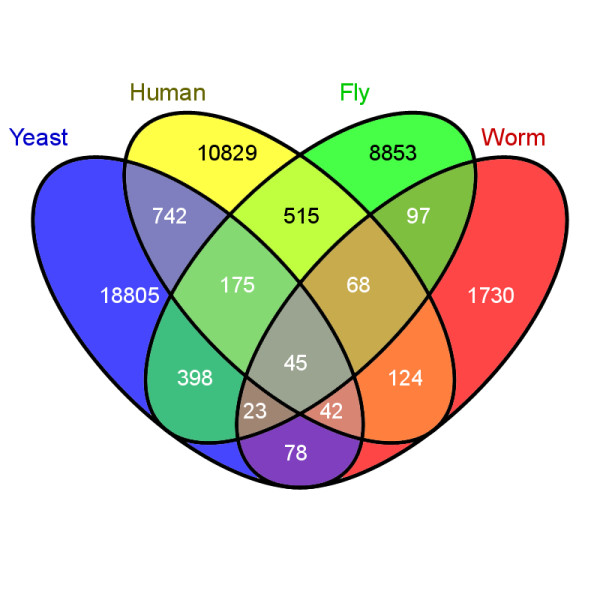
**A family-oriented overview of the input PPIs**. A 4-way Venn diagram illustrating the overlap of PPI evidence between four of the considered seven species: human, yeast, fly and worm. Each cell in the diagram is labeled with the number of pairs of protein families for which members interact in the corresponding species. For example, there are 742 pairs of protein families such that in both yeast and human there exists at least one interaction between members of the two families and no such interactions exist for fly and worm. Only about 0.5% (42514 of 8280415) of possible family pairs we consider have any evidence for interaction in any of the four species. Of these only 0.1% (45 of 42514) have evidence in all four species, which seems small, given that all considered families are evolutionarily conserved. However, the size of the overlap presumably corresponds to the fraction of the interactomes sampled experimentally, rather than to the actual level of conservation. For example, while there is a significant size difference between the overlap of the relatively best sampled yeast and human interactomes (742+175+45+42 = 1004 family pairs) and the overlap between yeast and worm interactomes (23+45+42+78 = 188 family pairs), the fraction of family pairs with PPI evidence from human and worm overlapping with such pairs in yeast is of the same magnitude (8% and 9%, respectively). It is highly probable that many of the homologous interactions in yeast and human have, yet unidentified, counterparts in worm and similarly in the other species. CAPPI uses phylogenetic information and probabilistic modeling to identify the most probable interactions in each species given the joint evidence from all input datasets and considering their reliability.

We develop a comprehensive framework for integrating and transferring PPI evidence across species. Our approach combines and extends the concepts of interlog mapping and Bayesian data integration. As opposed to the interlog approach, we employ PPI evidence from all homologous proteins, instead of using only the best-matching sequences in each case. This strategy is advantageous given the sparseness of the source datasets from which new interactions could be inferred. It is also motivated by the fact that the role of an individual protein in one species may be distributed over several homologous proteins in another species. Further, we use a Bayesian modeling framework to integrate PPI evidence from diverse experimental sources, taking into account their reliabilities and coverage. The evidence is accounted for in the context of the families' phylogenetic trees and under an assumed model of network evolution, which assigns probability scores to events of interaction loss or gain following a duplication or a speciation event (duplication and divergence model). Intuitively, the closer a given pair of proteins is to another pair, the more impact the evidence for one pair has on predicting the interaction of the other pair. The amount and reliability of the evidence, as well as the evolutionary proximity of the observed interactions to the pair of proteins in question, determines the posterior probability of interaction computed by our framework. We apply CAPPI to infer protein-protein interactions in seven eukaryotic species: human (*H. sapiens*), mouse (*M. musculus*), rat (*R. norvegicus*), fly (*D. melanogaster*), worm (*C. elegans*), yeast (*S. cerevisiae*), and thale cress (*A. thaliana*). The initial steps of our analysis preprocess the data and gather experimental evidence for interaction between members of distinct protein families. To this end, we identify groups of homologous proteins by clustering all non-redundant protein sequences downloaded from the Integr8 database [41] and pull relevant PPI data from IntAct [42], MINT [43] and DIP [44] databases (see Additional file 1 for details). The family-oriented view of the overlap of available PPI evidence for four best-represented interactomes is shown in Figure 1.

We consider two modes of application of our framework. First, the integration mode which gathers all available input data to provide a reconciled interactome view for each species. Second, the prediction mode which predicts the interactions for each species only based on the evidence from the other species (blind test). To demonstrate the different aspects of our method and enable a straight-forward comparison to the previous approaches we use different combinations of the input datasets and different reliability values, yielding the following sets of inferred interactions (for details see Additional file 1):

**CAPPI-Integ**: interactions for all seven species inferred using all available experimental datasets.

**CAPPI-Integ-3sp**: yeast, fly and worm interactions inferred based on experimental datasets from Ito *et al*. [2], Uetz *et al*. [1], Giot *et al*. [45] and Li *et al*. [46], with reliability parameters set according to [33].

**CAPPI-Pred**: interactions inferred for each species using experimental datasets only from the other six species.

We compare the results of CAPPI with the following methods:

**Domain-ML**: a maximum likelihood domain-oriented method [33]. Yeast interaction predictions, based on experimental datasets of Ito, Uetz, Giot and Li, were provided by the authors.

**Interlog**: an interlog-based method implemented in [47]. The program was downloaded from the InteroPORC website http://biodev.extra.cea.fr/interoporc/Default.aspx and ran for each species using experimental datasets only from the other six species (same datasets as in CAPPI-Pred).

In the following sections, we investigate the performance of our method on large-scale data, as well as in small-scale experiments focused on specific functional modules.

### Integration of interactions in seven eukaryotes

CAPPI-Integ provides an integrated and reconciled view of seven eukaryotic interactomes. Our ultimate goal is to provide a higher quality interactome for each input species. To assess the potential improvement, we perform two separate evaluations using a GO-based functional similarity measure and gold standard reference datasets.

### GO-based scoring

**Figure 2:**
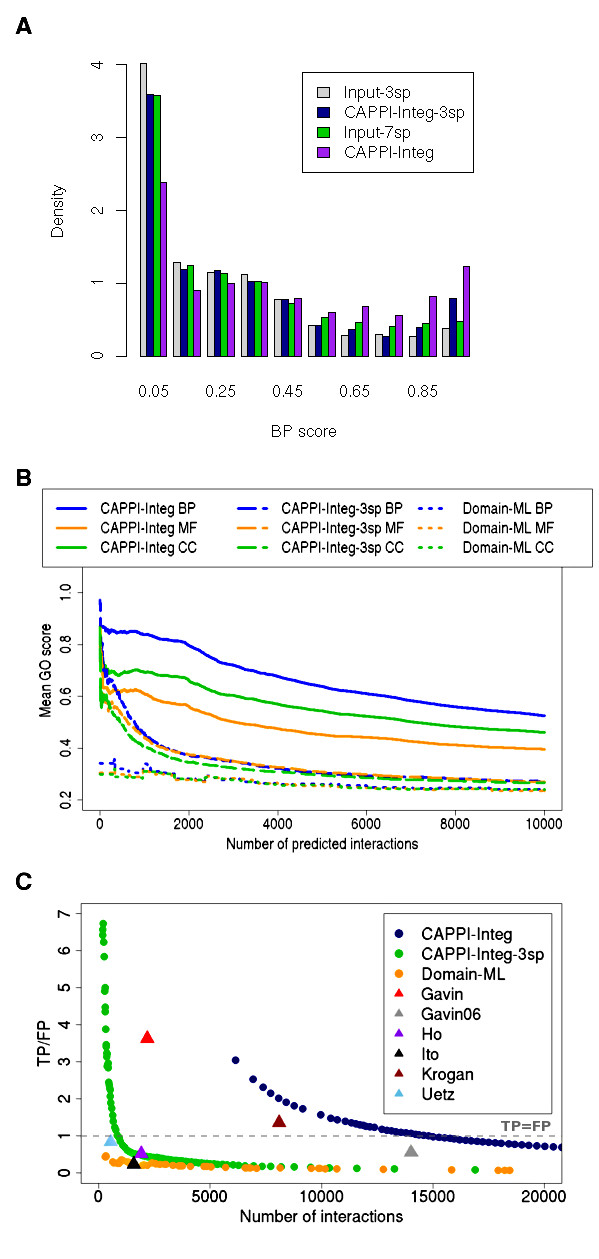
**Assessment of CAPPI-Integ predictions**. Assessment of CAPPI-Integ predictions. (A) Histogram of BP scores for the fly input datasets (combined) and corresponding inferred datasets of the same size (4049 PPIs in case of Input-3sp and CAPPI-Integ-3sp and 12107 PPIs in case of Input-7sp and CAPPI-Integ). Both CAPPI-Integ and CAPPI-Integ-3sp provide higher-scoring interactomes compared to their input datasets demonstrating the method's ability to use the interactions from distant species to make high quality predictions in other species. (B) Assessment of predicted yeast interactions using the three *GO scores*. The similarity of GO annotations of each pair of interacting proteins is measured in each ontology: biological process (BP), molecular function (MF) and cellular component (CC). CAPPI and Domain-ML predictions are ranked by their probabilities and the average GO score for the top *n *predictions is shown. CAPPI-Integ-3sp outperforms the domain based approach trained on the same experimental data. CAPPI-Integ integrates all available data from the seven species and further improves the predictions for yeast. (C) The ratio of true positives (TP) and false positives (FP) as a function of the number of yeast interactions. An interaction is deemed true positive if it is found in the reference dataset comprising co-complex and binary PPIs, and false positive, if the two proteins are assigned different localizations in the MIPS sub-cellular localization catalog (see text). The TP/FP ratios for the CAPPI-Integ, CAPPI-Integ-3sp and Domain-ML predictions are compared with the scores of the input experimental datasets. The gray dashed line marks the level at which the number of true positive predictions is equal to the number of false positive predictions.

Gene Ontology (GO) annotations are often used as indirect evidence for interaction. Intuitively, the more similar are the annotations of two proteins, the more confident we are in predicting an interaction between them. We first consider the biological process (BP) annotations and score our predictions, as well as the interactions from the input datasets, using the functional similarity measure from [48]. Mean *BP *scores for the input datasets and for the equal in size prediction datasets are summarized in Table 1. The scores of self interactions (present both in the input and in the inferred datasets) are excluded as they could introduce bias to the results (the GO annotations are identical in this case). Also, to avoid possible bias caused by the specific choice of proteins, input datasets are limited to interactions between members of conserved protein families used by CAPPI (see Additional file 1). For each CAPPI version in Table 1 we indicate the mean *BP *scores for the input dataset and the inferred output dataset of equal size. For example, in case of CAPPI-Integ the input yeast dataset contains 28590 interactions, for which the average *BP *score is 0.377. The corresponding CAPPI-Integ score of 0.412 was computed by taking the mean *BP *score of the 28590 best predictions in yeast (i.e. interactions with the highest probability). For each of the species CAPPI predictions receive significantly higher mean *BP *scores than the datasets used for training. The most significant improvement over the input datasets is achieved in case of the y, worm and rat predictions. The mean *BP *score for the entire fly input dataset is 0.295, while the CAPPI-Integ dataset of the same size achieves a mean score of 0.425 (44% higher). In case of worm and rat prediction we observe a 29% and 30% increase in the *BP *score, respectively. Our results show that CAPPI is able to produce reconciled interactomes which significantly outperform the input interactomes (see also Wilcoxon test *p*-values in Table 1). A detailed view of the distributions of *BP *scores for experimental and predicted datasets of protein interactions in *D. melanogaster *is presented in Figure 2A. The predicted datasets (both CAPPI-Integ and CAPPI-Integ-3sp) contain a lot more high-scoring interactions than are present in the input datasets. Interestingly, while the Input-3sp for fly is almost as good as the Input-7sp, CAPPI-Integ-3sp is significantly outperformed by CAPPI-Integ. This is largely due to the integration of additional high quality datasets from other species, from which CAPPI-Integ can transfer new evidence when inferring the fly interactome.

**Table 1:** *BP *score improvement over the input datasets.

Species	CAPPI-Integ				CAPPI-Integ-3sp			
	
	Data Size	Input Score	Output Score	Wilcoxon *p*-value	Data Size	Input Score	Output Score	Wilcoxon *p*-value
Yeast	28590	0.377	0.412	1.21e-31	1890	0.320	0.381	8.03e-06

Fly	12107	0.295	0.425	1.26e-113	4049	0.255	0.303	4.78e-05

Worm	2604	0.364	0.469	1.50e-21	856	0.374	0.485	2.02e-09

Arabidopsis	1349	0.596	0.623	0.02	NA			

Rat	1271	0.296	0.384	9.07e-06	NA			

Mouse	2456	0.417	0.463	1.53e-06	NA			

Human	17672	0.353	0.395	1.38e-31	NA			

The mean *BP *score of the input dataset and the inferred dataset of the same size are given for CAPPI-Integ and CAPPI-Integ-3sp. In all cases the inferred interaction set receives a significantly higher score than its input counterpart.

The improvement in mean *BP *score described above is achieved for relatively large predicted datasets (as large as the initial inputs). As we show in Figure 2B, *BP *scores are actually higher for our top predictions. Figure 2B plots mean similarity scores according to all three ontologies: biological process (BP), molecular function (MF) and cellular component (CC), as functions of the number of predicted interactions. The mean scores for both CAPPI versions are negatively correlated with the size of the output dataset. This enables the user to trade size for quality, obtaining a smaller dataset, but of greater reliability.

### Testing against gold standard datasets

We further survey the performance of our method using a set of gold standard binary PPIs pulled from [49] and [8], as well as co-complex data from the MIPS [50] and CYC2008 [51] complex catalogues (see Additional file 1 for details). Once again, we score CAPPI predictions and compare them to the scores of the input datasets. The results are presented in Figure 2C. The figure plots the ratio of true positive and false positive interactions present among a subset of a given size. The true positive interactions are either confirmed by binary PPIs or known to participate in a characterized complex. Unfortunately negative gold standard sets of non-interacting protein pairs are not available. We take a standard heuristic approach and consider pairs of proteins with different subcellular localization as putative negative examples. We note that in certain situations, e.g. signalling pathways, it is possible that interacting proteins are in fact in different cellular compartments. Note also that in general true interactions constitute only a very small fraction of all possible protein pairs - at most 0.5% in yeast based on recent estimates from [7]. This is reflected in our reference datasets. The positive reference used in this case contains 22480 PPIs and co-complex pairs while the negative set contains 4857065 differencially localized pairs (see also Additional file 1). It is unlikely to identify a true interaction by pure chance alone. Results presented in Figure 2C confirm the previous observation that reliable interactions are generally ranked high by our method. It is comforting that both CAPPI datasets contain more confirmed interactions than differentially localized pairs among the top ranked predictions (TP/FP >> 1). Note that a reference interaction can only be identified if a relevant evidence interaction is present in the input experimental evidence for one of the species. Given that the gold standard datasets generally do not have a large overlap with the input high-thoughput datasets, many of the reference interactions will not be inferred by any integration procedure. Importantly as shown in Figure 2C CAPPI-Integ-3sp has a much higher TP/FP ratio than the input yeast datasets (Ito and Uetz) used for its training. CAPPI-Integ integrates four more high-throughput yeast datasets and consistently scores higher than three out of four of these inputs - Gavin (2002) dataset has a higher score, but for a smaller number of interactions.

### Prediction of interactions in a blind test

We continue the performance evaluation by testing CAPPI's ability to predict interactions in a blind test. To this end, we compute the CAPPI-Pred dataset by iteratively leaving out PPI data of one of the seven species and predicting its interactions based only on the data from the other six species. We discuss the assessment of yeast and human predicted interactomes based on the two scoring frameworks.

**Figure 3:**
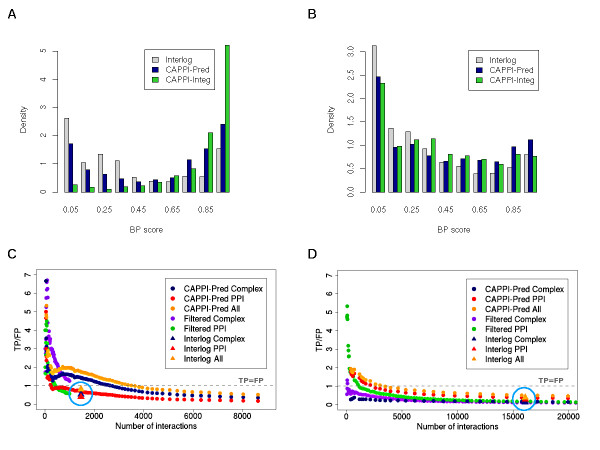
**Assessment of CAPPI-Pred predictions**. Assessment of CAPPI-Pred predictions. (A) and (B): Histograms of BP scores for the predicted yeast (A) and human (B) PPI datasets of the same size (1576 yeast PPIs and 17105 human PPIs) from the Interlog method, CAPPI-Pred and CAPPI-Integ. (C) and (D): The ratio of true positives and false positives as a function of the number of interactions in yeast (C) and human (D). An interaction is deemed true positive if it is found in the reference dataset of either co-complex interactions (Complex) or binary PPIs (PPI), or in any available reference set (ALL) and false positive, if the proteins are assigned different cellular localization (see text). Plots labeled as "Filtered Complex" and "Filtered PPI" show the results of selected CAPPI predictions which are part of dense clusters tested against either the co-complex reference (Complex) or binary reference (PPI). The gray dashed line marks the level at which the number of true positive predictions is equal to the number of false positive predictions.

Figure (3A and 3B) shows multiple histograms summarizing the *BP *score distribution among yeast and human predictions, respectively. The sizes of the predicted dataset (1576 for yeast and 17105 for human) have been selected to allow comparison with the interlog mapping predictions (see next section for details). Interestingly, we observe that while the performance of CAPPI-Pred is lower than CAPPI-Integ in case of yeast predictions, the opposite is true for the predicted human interactome. This suggests that while the yeast input interactions are necessary for good prediction results, human input datasets, on average, bring a less notable contribution.

In Figure 3C we plot the ratio of true positives and false positives as a function of the number of yeast PPIs returned by CAPPI-Pred. We evaluate the predictions separately using co-complex datasets (CAPPI-Pred Complex), gold standard binary PPI datasets (CAPPI-Pred PPI), as well as all available reference data (CAPPI-Pred All) - see Additional file 1 for details. An analogous study is performed for the predicted human interactome using the HPRD (complex and binary PPI) catalogues as reference (see Figure 3D). Note that similarly as for yeast, also for human the positive reference set is significantly smaller than the negative reference set. The joint human reference set (All) contains 57,093 protein pairs, which is less than 0.2% of the number of differentially localized pairs - consistent with the expected ratio of true interactions to all protein pairs in human, as estimated in [52]. The results show that CAPPI is able to infer high-scoring PPIs also in the case when no interactions from the predicted interactome are included in the training set. Most of the top predictions are confirmed by experimental data. We observe that while more yeast predictions are confirmed by co-complex pairs than by binary PPI data, the opposite is true in case of the human predictions. This can be explained by the differences in size of the respective reference datasets for the two species (see Additional file 1). When all available reference data is considered (CAPPI-Pred-All), the TP/FP ratios for the top 5,000 interactions in yeast and human are comparable (~0.8).

### Filtering co-complex predictions

Evolutionary pressures are more likely to constrain essential functional units than individual interactions [36]. Thus co-complex PPIs should be easier to map accurately across species. This premise was previously explored in [38], where the authors showed that screening PPI predictions against conserved clusters improves prediction specificity. In an attempt to increase the percentage of co-complex PPIs in our predictions, we filtered the CAPPI-Pred output dataset, leaving only the predicted PPIs placed within conserved dense network regions. To this end, an ancestral interaction network was computed as in [39], and clustered using the MCL algorithm [53] to identify dense clusters. Each cluster was projected onto the network of the extant species (yeast or human) and CAPPI-Pred predictions within the projected regions were identified as a result. As shown in Figure (3C and 3D), this procedure significantly boosts the TP/FP ratio for both yeast and human data (see "Filtered Complex" plots). Interestingly, while the fraction of co-complex PPIs was increased, the fraction of confirmed binary PPIs was in general lowered by the filtering (except for the top ranked human predictions), suggesting that many binary PPIs placed outside or between protein complexes are filtered out in this case. This is in line with the observations made in [8] that binary and co-complex datasets are of complementary nature and often have small overlap.

### Comparison with previous high-throughput multi-species approaches

Numerous existing computational approaches for predicting protein associations in multiple species can be loosely divided into three categories. The first group of methods contains approaches for predicting interactions *de novo *from protein sequence. These methods often utilize evolutionary information such as phylogenetic profiles or gene fusion events, but they do not explicitly transfer pre-identified interactions from one species to another. The second group of methods takes as input experimentally identified PPIs, integrates them and transfers the evidence to other species. The third group of studies is directed towards integration of heterogeneous experimental evidence such as PPI, mRNA co-expression, phylogenetic profile similarity, co-localization, domain associations, etc., and attempts to predict various types of functional associations, not limited strictly to protein-protein interactions. CAPPI was specifically designed as a model-based approach for integrating and transferring protein-protein interactions across species and as such it falls into the second category. Here we compare the performance of our method and two well-established frameworks for mapping PPIs: the interlog approach and the domain-based maximum likelihood method.

### Comparison with the domain-based maximum likelihood approach

In [33] the domain-domain interaction prediction method was generalized to multiple species and applied to infer interactions in yeast, worm and fly (we refer to this method as the Domain-ML approach). As a final output, this approach predicts protein-protein interactions based on inferred interactions between conserved domains. Liu *et al*. trained their method using Ito, Uetz, Giot and Li experimental datasets, so the their results can be directly compared to CAPPI-Integ-3sp. Note that only the yeast interaction predictions were provided by the authors. The mean *GO *scores for Domain-ML and CAPPI are shown in Figure 2B. CAPPI-Integ-3sp significantly outperforms Domain-ML in terms of all three *GO *scores. The performance evaluation using gold standard data (Figure 2C) also indicates a higher accuracy of CAPPI compared to the domain-based approach.

### Comparison with the interlog-based approach

Next, we compare our results with a popular method of interlog mapping. This approach, similarly to CAPPI, relies on protein sequence similarity to transfer the interaction evidence across species. We choose for comparison the interlog mapping implementation from [47] and use the same input data for predicting our CAPPI-Pred dataset (for details see Additional file 1). Figure (3A and 3B) provides the distributions of *GO *scores for the Interlog and CAPPI datasets of the same size: 1576 (yeast) and 17105 (human), respectively. CAPPI predictions also contain a larger fraction of highest-scoring interactions (those with *GO *score > 0.8) and obtain a higher average score. The mean score for the CAPPI-predicted yeast dataset is noticeably higher than that of the Interlog method (0.57 vs. 0.39). CAPPI's advantage is also apparent in case of the human predictions (mean score 0.42 vs. 0.33). To assess the significance of the difference in score distributions we performed the Wilcoxon test which returned *p*-values < 2.2 × 10^-16 ^in all cases.

Figure (3C and 3D) shows the mean scores for the Interlog output (in blue circles), which can be compared with the CAPPI rankings. In all cases CAPPI achieves a higher fraction of true positive interactions: 0.88 vs. 0.47 for the yeast co-complex predictions, 0.72 vs. 0.40 for the yeast binary PPI prediction, 0.16 vs. 0.14 for the human co-complex predictions, and 0.38 vs. 0.28 for the human binary PPI predictions. As we show in the next section, CAPPI recovers many known interactions within essential functional modules enabling the reconstruction of module subunits. The InteroPORC method is too restrictive in most of the studied cases (see Additional file 1: Table S1), suggesting that a less stringent ortholog search is needed. In fact this is recognised in [47] where more sensitive methods are considered for predicting interactions in cyanobacterium *Synechocystis*. An additional advantage of our method lies in the provided ranking (induced by the posterior probabilities), which enables the user to easily identify the most reliable interactions. As an example, for the purpose of selecting human PPI targets for verification, one could make a heuristic decision to consider only around 3,500 top predictions for which the TP/FP ratio is greater than 1 (see Figure 3D).

### Case studies: mapping interactions within conserved functional modules

We now zoom-in on specific examples of functional units in the interactomes of human, yeast and thale cress, and analyze co-complex interactions inferred by CAPPI-Pred. In all described cases we demonstrate that the general topological features and organization of these complexes, as well as many known pairwise PPIs, can be recovered by our method based solely on data from the other species. We verify the inferred interactions against previously reported experimental data and assess the significance of our predictions. For an example of how the threshold selection impacts the number of interactions and the resulting *p*-value see Additional file 1: Figure S1. Note that in the following discussion gene names are used to denote corresponding proteins.

### Human and yeast proteasome subnetworks

The ubiquitin-proteasome pathway is essential for eliminating damaged proteins and for regulation of intra-cellular level of proteins involved in wide spectrum of cellular functions [54]. It is conserved in eukaryotes, from yeast to human. The 26S proteasome complex contains a 20S catalytic core particle (CP), which is capped on each side by a 19S regulatory particle (RP). The structure of the 20S proteasome from yeast has been resolved [55]. It consists of 28 protein subunits: two *α*-rings (*α*1,...,*α*7) and two *β*-rings (*β*1,...,*β*7). The 19S proteasome can be further decomposed into two subcomplexes: the base (Rpt1-Rpt6, Rpn1, Rpn2, Rpn10 and Rpn13 - the last one probably not present in human) that binds directly to the 20S proteasome, and the lid (Rpn3, Rpn5-Rpn9, Rpn11, Rpn12 and Sem1), which is a peripheral subcomplex. In addition there is a number of transiently associated factors like p27 and S5b (the latter is apparently not present in yeast). We discuss our predictions of the 26S proteasome interactions from yeast and from human separately.

**Figure 4:**
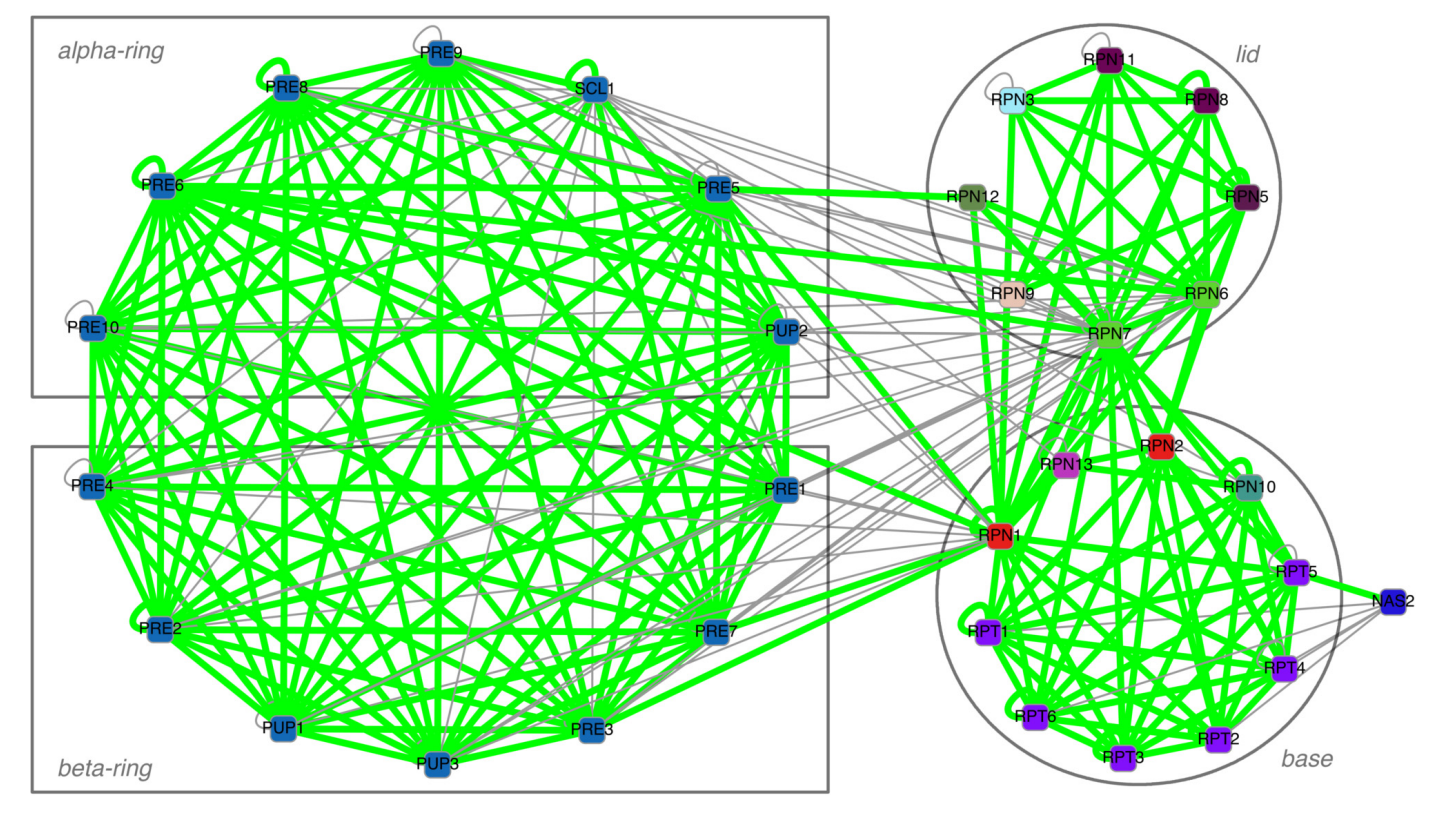
**Inferred PPIs within the yeast proteasome complex**. Interaction network of the yeast 26S proteasome complex as inferred by CAPPI-Pred. Nodes represent gene products and node colors represent protein families identified by sequence clustering. 177 of the predicted interactions which have been previously detected experimentally are denoted by green edges. 66 other PPI predictions are denoted by gray edges. The *p*-value of the predicted network is 4.348 × 10^-16^. The networks are visualized using the Cytoscape software [73].

Predicted interactions in the yeast 26S proteasome are depicted in Figure 4. Overall, at the selected threshold we identify 177 confirmed interactions and 66 unconfirmed ones. The graph inferred by CAPPI is split into four parts that correspond to the four subcomplexes of the proteasome: *α*-ring, *β*-ring, lid and base. The *α*-ring and the *β*-ring have a dense set of interactions. Both of them together form a clique (i.e. every two proteins are predicted to interact), with most of the interactions being supported by experimental data. The lid and base are also very well represented and connected by 16 interactions, all of which are confirmed by previous experiments. We observe also the central role of Rpn7, which is predicted to interact with every subunit in the *α*- and and in the *β*-ring, as well as with six proteins in the lid subcomplex and eight in the base. Another hub protein identified is Rpn1, which has twelve interaction partners among the alpha and beta proteins (four of which are confirmed), seven partners in the base and seven in the lid (all having experimental support). On the other hand, the transiently associated NAS2 (p27) is predicted to interact only with the AAA-ATPase subunits (Rpt1-Rpt6) of the base subcomplex. In general, interactions within the core subcomplexes of the yeast 26S interactome are accurately recovered based solely on data from other six species, demonstrating a high level of conservation of these PPIs. The vast majority of the 66 unconfirmed predictions are localized between the characterized subcomplexes. In fact only 7 of the 44 predicted interactions between the 20S catalytic core and and the 19S regulatory particles are backed by experimental evidence in yeast. The absence of experimental data for these PPIs in *S. cerevisiae *might be explained by insufficient coverage of the yeast interactome or by possible rewiring events which changed the topology of interactions between the conserved core subunits across species. The discussion of human proteasome PPI predictions is presented in Additional file 1.

### Human and yeast endosome subnetworks

The ESCRT complexes comprise a major pathway for the lysosomal degradation of transmembrane proteins (see [56]). We investigate the predicted interactions for the ESCRT complexes in human and yeast and compare the obtained results with the interactions reported in the literature. The list of proteins involved in these complexes was taken from [56].

**Figure 5:**
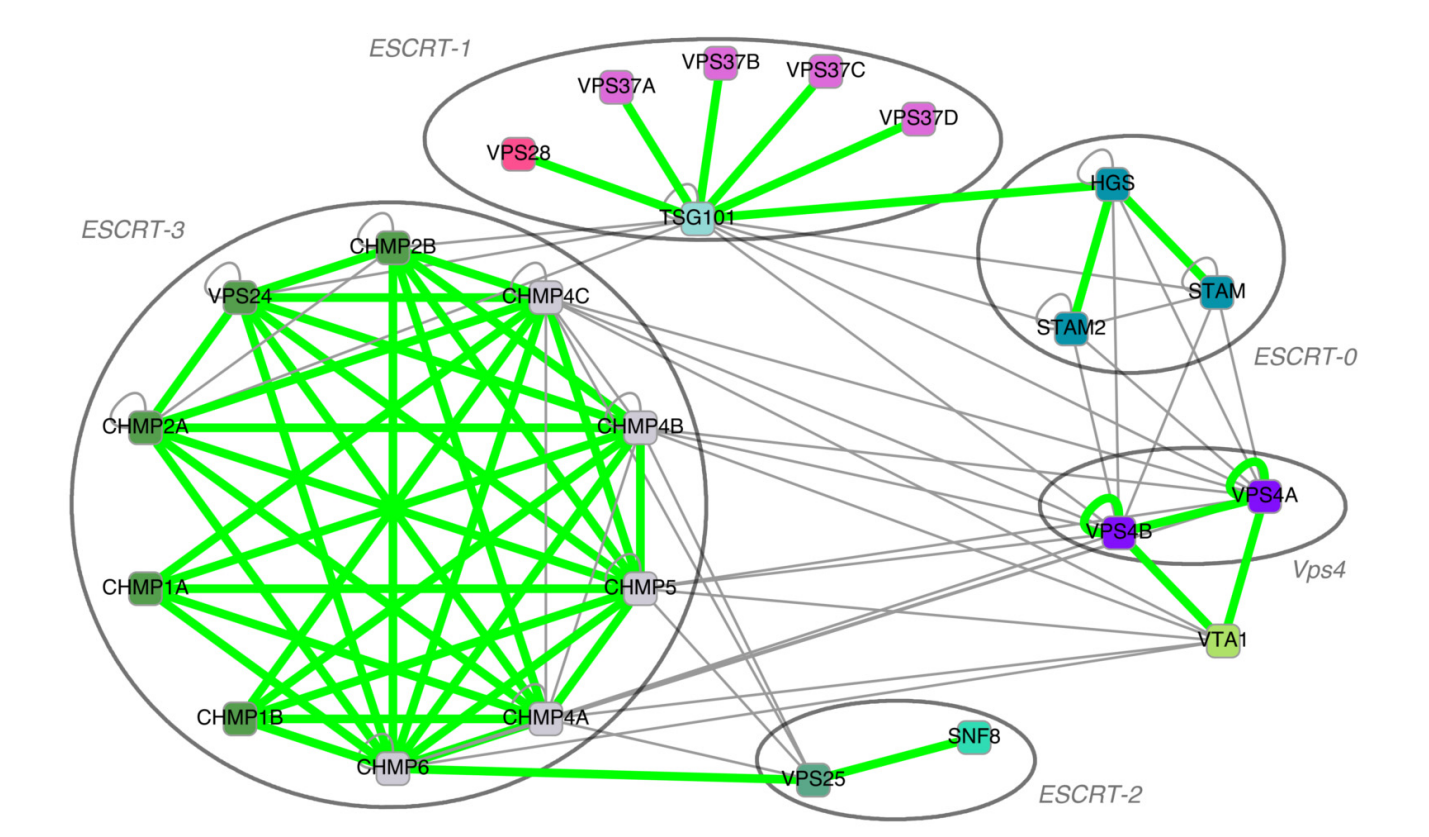
**Inferred PPIs within the human endosome complex**. Interaction network of the human endosome complexes as inferred by CAPPI-Pred. Nodes represent gene products and node colors represent protein families identified by sequence clustering. 49 predicted interactions which have been previously detected experimentally are denoted by green edges. 49 other PPI predictions are denoted by gray edges. The *p*-value of the predicted network is 3.977 × 10^-9^.

Human ESCRT co-complex interactions as predicted by our method are depicted in Figure 5. CAPPI-Pred was able to recover all five complexes discussed in [56]. These complexes are: ESCRT-3 (well represented as a dense connected component with most edges reported in previous experiments), ESCRT-1, ESCRT-0, the Vps4 complex, and the ESCRT-2 complex. Interestingly, our results suggest that proteins CHMP1B and CHMP5 should be assigned to the ESCRT-3 complex. This association of CHMP1B and CHMP5 (consistent with the so called 'CHMP nomenclature') has been recently proposed in [57]. Moving on to the right side of the graph, we notice that the VPS4 proteins together with protein VTA1 form a triangle comprising of three reported interactions. A similar observation can be made for the ESCRT-0 complex (HGS, STAM1 and STAM2), except that the interaction STAM-STAM2 is not supported by previous experimental data. Also, the topology of interactions presented in Figure 5 suggests an important role of the TSG101 (mammalian VPS23) protein, which joins ESCRT-1 with three other complexes (ESCRT-3, ESCRT-0 and Vps4). TSG101 also takes part in five identified interactions within the ESCRT-1 complex, all of which have backing experimental evidence in human. Please refer to Additional file 1 for the discussion of yeast ESCRT complex predictions.

### Human mRNA decay complexes

**Figure 6:**
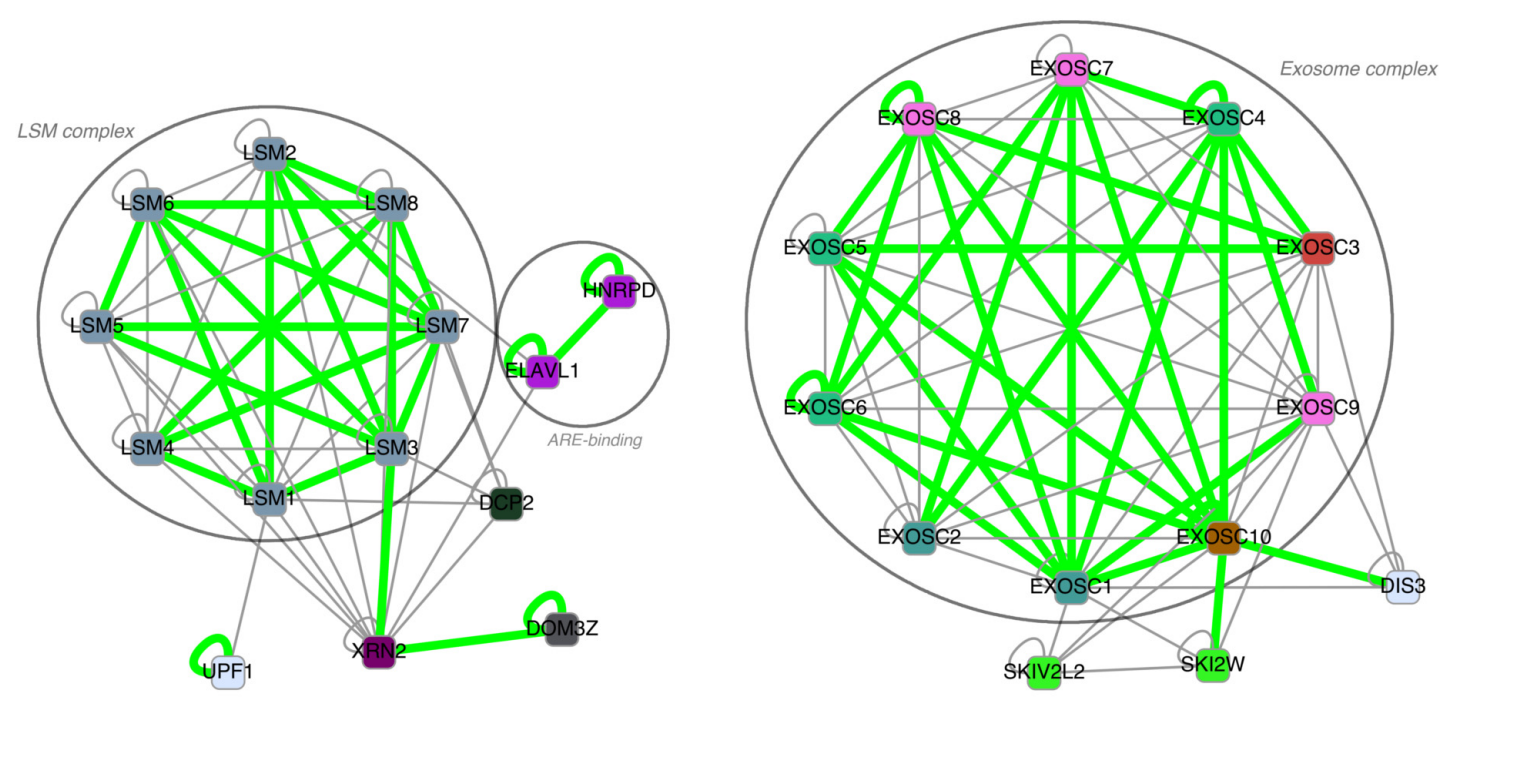
**Inferred PPIs within the human mRNA decay complexes**. Interaction network of the human mRNA decay complexes as inferred by CAPPI-Pred. Nodes represent gene products and node colors represent protein families identified by sequence clustering. 53 of the predicted interactions which have been previously detected experimentally are denoted by green edges. 76 other PPI predictions are denoted by gray edges. The *p*-value of the predicted network is 1.868 × 10^-15^.

Next we investigated CAPPI's interaction predictions between proteins involved in human mRNA degradation (see [58]). The subgraph of predicted interactions is presented in Figure 6. We have a very good coverage of the human exosome complex represented by six RNase PH domain subunits (EXOSC4 (Rrp41), EXOSC5 (Rrp46), EXOSC6 (Mtr3), EXOSC7 (Rrp42), EXOSC8 (Oip2), EXOSC9 (PMScl-75)), three S1 RNA-binding domain subunits (EXOSC1 (Csl4), EXOSC2 (Rrp4), EXOSC3 (Rrp40)), and an RNase D-like subunit EXOSC10 (PMScl-100). This complex comes out as a complete subgraph (a clique) with no interactions with the other two complexes. The role of most of the subunits of the complex, in terms of interacting partners, is quite comparable. One of the exceptions is the EXOSC9 (PMScl-75) protein which is the only RNase PH domain subunit predicted to interact with DIS3 and two helicases (SKI2W and SKIV2L2). Other exosome complex members interacting with DIS3 are S1 RNA-binding subunits EXOSC1 (Csl4) and EXOSC3 (Rrp40), as well as EXOSC10. EXOSC1 and EXOSC10 also have predicted interactions with helicases SKI2W and SKIV2L2. In general, data on interactions of the peripheral subunits with the exosome complex are scarce, as reported in [58], which makes our predictions a potentially valuable target for experimental verification. The second complex which comes out as a dense subgraph in our network is the LSM complex. It consists of eight proteins (LSM1-8), forming a clique of predicted interactions, many of which are confirmed experimentally (see [58] Figure 3A). The two proteins with the largest number of confirmed interactions within the complex are LSM3 and LSM7. Both of these proteins have confirmed PPIs with six out of seven other LSM members (additional PPIs predicted by our method are LSM3-LSM4 and LSM7-LSM1). The third complex which can be retrieved from the network in Figure 6 consists of two AU-rich element ARE-binding proteins (ELAVL1 (Hur) and HNRPD (Auf1)). All three interactions predicted inside this complex are confirmed by recent experimental data (see [59]). Among the unverified predictions is an interaction of this complex with the LSM complex (via LSM2) and with another mRNA decay factor XRN2.

### A. thaliana SWI/SNF chromatin remodeling complex

**Figure 7:**
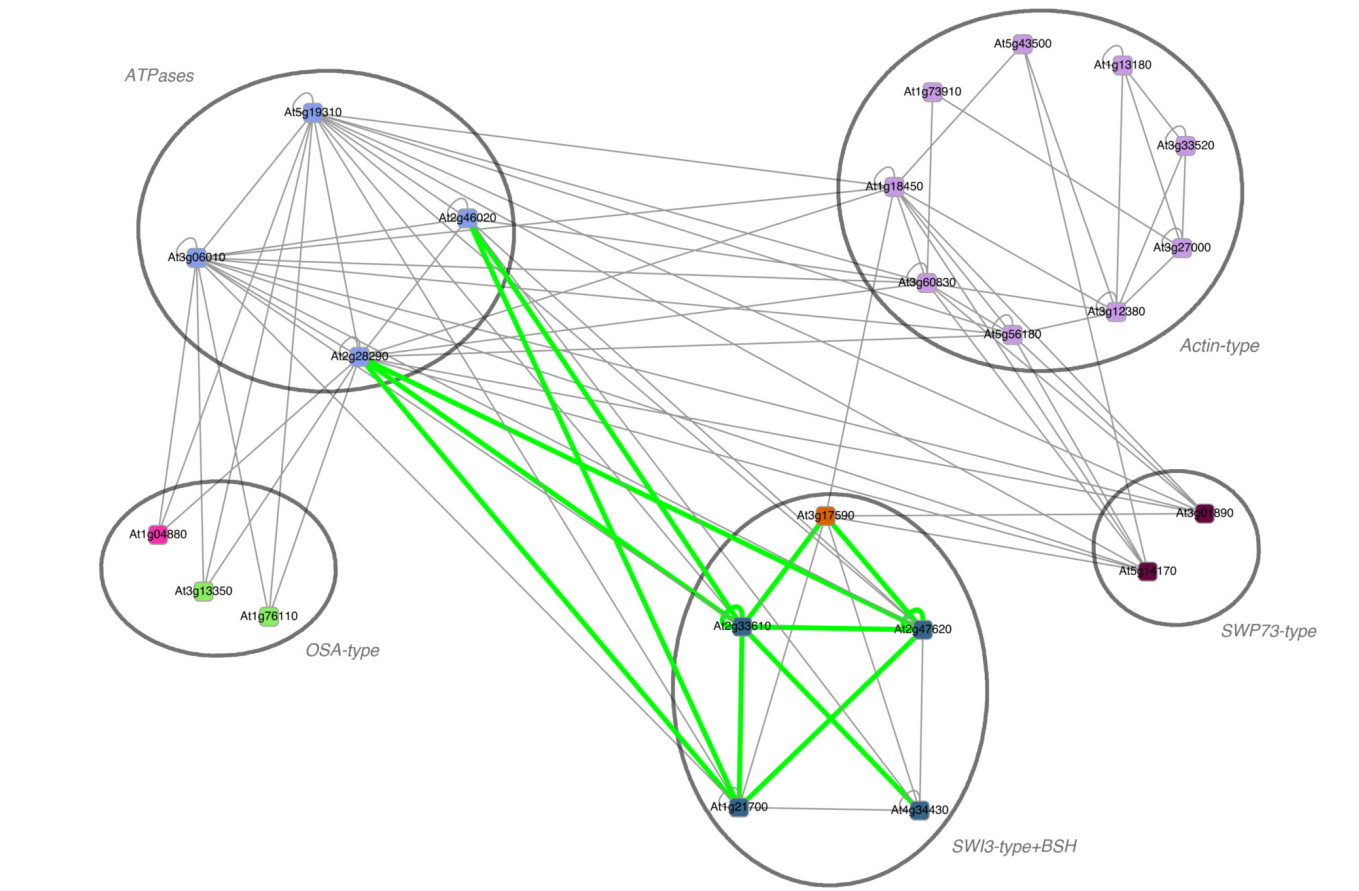
**Inferred PPIs within the A. thaliana SWI/SNF chromatin remodeling complex**. Interaction network of the putative SWI/SNF complex in Arabidopsis as inferred by CAPPI-Pred. Nodes represent gene products and node colors represent protein families identified by sequence clustering. 13 of the predicted interactions which have been previously detected experimentally are denoted by green edges. 83 other PPI predictions are denoted by gray edges. The *p*-value of the predicted network is 6.381 × 10^-10^.

In yeast and mammals, ATP-dependent chromatin remodeling complexes belonging to the SWI/SNF family play an essential role in the regulation of transcription. In Arabidopsis chromatin remodeling complexes are known to a much smaller extent. No plant SWI/SNF complex has been established and characterized to date, but it is highly probable that such complexes exist in plants (see [60]). For this reason it seems desirable to employ a computational approach for predicting interactions in the plant SWI/SNF putative complex and generate plausible working hypothesis. We present a zoom-in view of the SWI/SNF putative complex in Figure 7. A larger zoom-out view containing other homologs of the putative SWI/SNF complex members is presented in Additional file 1: Figure S2.

The graph in Figure 7 contains the core SWI/SNF proteins - the SWI3-type proteins: At2g47620 (SWI3A), At2g33610 (SWI3B), At1g21700 (SWI3C), At4g34430 (SWI3D), together with the SNF5-type protein At3g17590 (BSH). This core is presented at the bottom of the graph. In addition to the above proteins we considered four groups of Arabidopsis proteins which are reported to play a putative role in chromatin remodeling in this plant (see [60]). These are: four ATPases which are reported in [60] as potential members of the SWI/SNF complex (At2g46020 (BRM), At2g28290 (SYD), At3g06010 (Chr 12), At5g19310 (Chr 23)); two SWP73-type proteins (At3g01890 (SWP73A), and At5g14170 (SWP73B)); nine actin-related proteins (At3g27000 (ARP2), At1g13180 (ARP3), At1g18450 (ARP4), At1g73910 (ARP4A), At3g12380 (ARP5), At3g33520 (ARP6), At3g60830 (ARP7), At5g56180 (ARP8) and At5g43500 (ARP9)); and three OSA-type proteins (At1g04880, At1g76110, and At3g13350). We excluded from the graph proteins which did not show any predicted interactions. Altogether we identified 13 of 14 known interactions between the proteins visualized in Figure 7 - the missing one is At3g01890-At1g21700 (see [60]). We notice some interesting peculiarities of the presented network. Three of four of the SWI3-type proteins, are predicted to interact with the four ATPases. Only one actin-type protein (At1g18450) has a predicted interaction with the SWI/SNF core and only two more (At3g60830 and At5g56180) can be associated with the complex through member ATPases. The ability to make distinctions within homologous groups is an important feature of our approach. While methods mapping interactions to highly similar orthologs usually make very specific predictions and avoid false-positives, they are also likely to miss many true interactions which can be inferred from slightly less similar proteins. As summarised in Additional file 1: Table S1, the restrictive search applied in InteroPORC fails to map the known interactions in the SWI/SNF complex in *A. thaliana*. In fact according to the PORC ortholog clusters, only two proteins (SWI3C and SWP73A) have orthologs in any of the other six eukaryotic species considered here. In this case, a less stringent method is clearly needed. On the other hand, CAPPI bases its prediction on evidence from all homologs and thus is in danger of loosing sensitivity and assigning the same interactions to all family members. The above examples demonstrate that we can avoid these potential pitfalls by considering family members in phylogenetic context when integrating and distributing the interaction evidence.

These observations are strengthened when we consider the larger family-oriented view of the SWI/SNF-related network in Additional file 1: Figure S2. This graph was obtained from the one in Figure 7 by expanding the set of proteins to all members of the considered protein families (once again, proteins without any interactions were removed). Interestingly, the four peripheral families represented in the graph can be divided into smaller subfamilies based on the interactions partners of their members. Specifically, of the 14 ATPases presented in the larger graph only the four above described are predicted to interact directly with the core of the SWI/SNF complex. Two of them (At2g46020 (BRM) and At2g28290 (SYD)) have confirmed interactions while for the other two (At3g06010 (Chr 12), At5g19310 (Chr 23)) interaction hypothesis based on sequence similarity were formulated [60]. In fact the entire ATPase family, as detected by our method, contains 48 Arabidopsis proteins (a vast majority not having any predicted interactions to other proteins in the SWI/SNF subnetwork), which makes the presented predictions even more significant. These specific cases of confirmed predictions let us suggest that some of the distinctive members of the other protein families predicted to interact with the putative SWI/SNF complex (At1g18450 and six OSA family members interacting with At3g17590, five SWP73 family members interacting either with At3g17590 or at least one of the SWI3-type proteins, as well as five other actin family members interacting with ATPases At2g46020 and At2g28290), may pose valuable targets for future experimental validation.

## Conclusion

We have presented a systematic phylogeny-based framework for reconciling PPI datasets across species and inferring missing interactions. Our method naturally incorporates interaction evidence from different species and experimental sources. It considers the reliability of each source and the evolutionary relationships between protein pairs. The approach was successfully applied to compute integrated interactomes for seven eukaryotic species, providing confidence scores for each possible edge in each network. Detailed analysis of our predictions indicates that we can accurately recover known interactions within conserved protein complexes. Confirmed interactions identified in a blind test provide a strong case for our top-ranked predictions, many of which await experimental verification. We also find that while core subcomplexes can be accurately recovered based solely on the data from distant species, many of the between-module interactions are harder to identify this way, suggesting possible rewiring events. One natural direction for future research is to extend our framework to include other kinds of data which may serve as indirect evidence of interaction. The integration of heterogeneous experimental sources with account of the phylogenetic model may possibly improve existing catalogues of functional associations.

## Methods

### Bayesian model of network evolution

**Figure 8:**
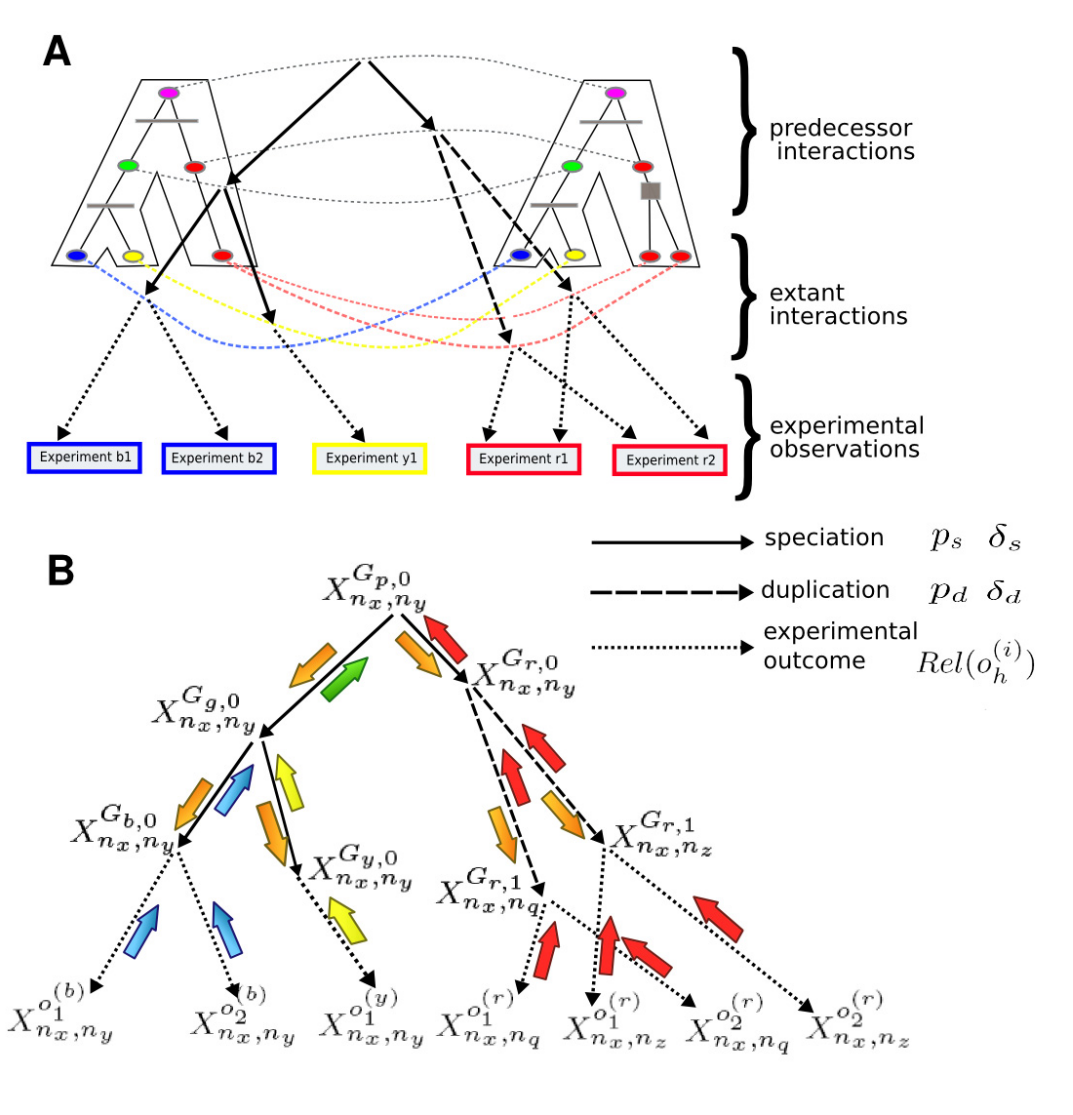
**Inferring protein interactions via message passing**. A toy example of the Bayesian tree model of evolution of interactions between members of two protein families for three species: blue, yellow and red. For each species a certain number of experimental datasets is given: two for blue and red and one for yellow. Part (A) shows two reconciled trees for the considered families together with putative protein interactions at each level of evolution. The proteins in the trees are represented by ellipses (with color corresponding to their species). The speciation events are marked by horizontal lines and the duplication events are marked by filled squares. The evolution of the ancestral interaction between the root proteins (purple) can be traced down the trees to the extant interactions. Evidence for the extant interactions can be found in the experimental datasets. In (B) a random variable is associated with each putative interaction. A solid arrow indicates a dependence between two random variables which comes from the speciation event. Similarly, a dashed arrow indicates a dependence for the duplication event. Finally, dotted arrows represent an interface between the true interactions in extant species and the observed experimental evidence. The parameters *p*_*s*_, *δ*_*s*_, *p*_*d *_and *δ*_*d *_determine the probability of retaining or gaining an interaction during evolution, while the reliability of each dataset (*Rel*()) determines the probability of identifying a true interaction or a false positive one. Arrows colored blue, yellow, red and green represent messages corresponding to interaction evidence coming from each of the species. These messages are passed up the tree in the first phase of the MP algorithm. In the second phase, messages containing aggregated evidence from one side of the tree are passed down to the other side (orange arrows).

We start by briefly recapitulating the network growth model from [39] which, given the ancestral network *G*_1,0 _determines the probability of interaction between proteins at every stage of evolution. The model has four parameters: *p*_*d*_, *δ*_*d*_, *p*_*s *_and *δ*_*s*_. It assumes that starting from the ancestral graph *G*_1,0 _a sequence of duplications and speciations is performed where these events are determined by reconciled phylogenetic trees precomputed for each protein family. We denote by *G*_*i*,*j *_= (*V*_*i*,*j*_, *E*_*i*,*j*_) the graph representing the protein network of *s*_*i *_after the *j*-th duplication event occurring in this species. In case of a node duplication event we replace the node by two copies. For each copy we retain each of its edges with probability *p*_*d *_and insert edges adjacent to the copy with probability *δ*_*d *_(independently for each copy and each edge). In case of a speciation event we make two copies of the network. In each network copy we retain each edge with probability *p*_*s *_and insert each non-existent edge with probability *δ*_*s *_(independently for each network and each edge). Assuming this model and the provided phylogeny of each protein family we construct a Bayesian network (BN) model of protein interactions at all levels of evolution. In this BN model the probability  of interaction between a pair of nodes *n*_*x*_, *n*_*y *_∈ *V*_*i*,*j*_, depends on the existence or lack of an edge between the protein pair being the direct evolutionary predecessor (either before speciation or duplication) of the pair (*n*_*x*_, *n*_*y*_) (see Figure 8). A detailed description of the model is available in [39].

### Integrating diverse experimental data

The above-described model captures the basic notions of protein network evolution. We previously assumed that the PPI data is free of error and complete and we used the model to make inferences about the ancestral interaction networks. However, due to experimental errors and incomplete sampling, the real interactions and non-interacting protein pairs are not certain. This implies that the experimental data should only be used as supporting evidence of putative interactions. To model this accurately in our framework we keep the random variables corresponding to extant interactions unknown and add another level of random variables corresponding to experimental evidence (see Figure 8A). The evidence in each experimental dataset is weighted by the dataset's reliability.

Let  be the extant protein interaction network of a present-day species *s*_*i *_(we assume that *m*_*i *_is the final duplication occurring in *s*_*i*_). Let  be the set of experimental datasets for species *s*_*i*_, where each  is the set of protein pairs confirmed to interact in the *h*-th experiment. Let *Rel*() be the fraction of elements in  believed to be true positives. Let  be the set of non-interacting protein pairs in the graph . For each experimental dataset  we denote by  a random variable which takes value 1 if interaction (*n*_*x*_, *n*_*y*_) is present in this dataset and 0 otherwise. For each pair of proteins (*n*_*x*_, *n*_*y*_) and each dataset  we set the probability of observing a true interaction to be equal the true positive rate of the experiment, and the probability of observing a false positive interaction equal the false positive rate of the experiment, as follows:

where by |*A*| we denote the number of elements in the set *A*. Now each experimentally observed interaction can be naturally incorporated into the BN framework. Similarly each pair not observed to interact in the considered experiment ((*n*_*x*_, *n*_*y*_) ∉ ) can be incorporated into the model with conditional probabilities corresponding to the false negative rate and true negative rate of the experiment (see Additional file 1 for details). The model can also be easily generalized to incorporate distinct reliability values for each single interaction.

### Inferring extant protein interactions via message passing

The integrated BN model, comprising all PPI edges from every level of evolution and from the experimental datasets, is used to infer protein interactions in the input species. Each random variable corresponding either to a possible interaction, or to a single experiment outcome, depends on exactly one random variable which denotes an edge (or non-edge) in the direct evolutionary predecessor in the first case, and in the network of an extant species in the second case. The considered BN model is a set of Bayesian trees, where each tree represents the joint distribution of the random variables corresponding to putative interactions (which descended from a single edge in the ancestral graph) and the associated experimental evidence (an example of such tree is shown in Figure 8B). The tree structure allows us to apply Pearl's message passing (MP) algorithm [61] to compute the exact posterior probability of interaction between proteins in extant species, in time linear to the number of random variables (see Figure 8B for an example and [61] or [62] for details). Specifically we determine the posterior probability of interaction *P*( = 1|*O*) for each pair of nodes (*n*_*x*_, *n*_*y*_) in each extant network , where *O *denotes all experimental datasets for all species.

### Assessing PPI predictions in large-scale studies

In general, the assessment of PPI predictions posses problems due to the limited number of "gold standard" interactions and the lack of negative test cases. Motivated by previous studies, we employ two scoring schemes to assess the quality of predicted PPIs, as well as those from the input datasets. The first one compares Gene Ontology (GO) annotations [63] of adjacent gene products and measures their functional similarity. Functional similarity is used as an indirect measure of interaction: the more similar the annotations of the two proteins are, the more confident we are in deeming an interaction between them. We apply a recent information content method [48], implemented in the SemSim R package by Xiao Gou: http://www.bioconductor.org/packages/2.0/bioc/html/SemSim.html, which extends the measures previously proposed by [64] and [65]. For each pair of proteins we individually measure the similarity of annotations in each of the three ontologies: biological process (BP), molecular function (MF) and cellular component (CC). This results in a *BP *score, *MF *score and *CC *score, respectively, each ranging from 0 (no similarity) to 1 (maximum similarity). When the context allows, we refer to each of these scores as a *GO *score of a pair of proteins.

Our second kind of quality assessment is based on a comparison with a reference dataset. We estimate the ratio of true positive interactions (predictions which are confirmed in a reference dataset) and putative false positive interactions (unconfirmed predictions for which the two proteins have disjoint cellular localizations). A similar procedure was applied in [29]. We use separate reference datasets for binary PPIs (direct physical interactions) and for co-complex PPIs (pairs of proteins co-occurring within the same complex). For details on the reference datasets and the localization data see Additional file 1. Note that the proper sensitivity and specificity measures are hard to estimate because the reference sets of positive interactions and negative protein pairs are not comprehensive. Due to interdependencies between interactions, implied by our model, cross-validation cannot be easily applied. Instead, we perform a blind test in which we leave out the data of one species and predict its interactions only based on the data from the other species.

### Assessing predictions in functional module case-studies

For small-scale functional module case studies we report all interactions predicted among a determined set of proteins for a selected threshold value. To assess the statistical significance of interaction predictions we compute a *p*-value based on the hypergeometric distribution, where confirmed interactions are regarded as successes and unconfirmed interactions are regarded as failures (Fisher's exact test). As the predictions are made by CAPPI-Pred which is trained without the use of the input datasets for the predicted species, we use the held out input data as a reference. Note that it is possible that some of the reference interactions are in fact false-positives - an inherent risk of using high-throughput data. In this particular test, however, we are interested in assessing the possibility to predict a significant portion of known PPIs (of which many are from high-throughput studies) by a mapping from other organisms. The reference set is further extended in each case by PPIs curated from specific publications characterizing interactions within the studied complexes. These are as follows: [66,67] for the 26S proteasome PPIs, [56,57] for the endosome-related PPIs, [58] for the exosome-related PPIs, and [68-72] for the SWI/SNF-related PPIs. Note that for *A. thaliana *there are no high-throughput datasets available, so all reference data for this species come from small-scale studies.

## Authors' contributions

The authors together conceived the study and analysed the results. JD implemented the framework, computed the integrated interactomes and analyzed the high-throughput experiments. JT provided initial analysis of the small-scale case studies. Both authors contributed to writing the manuscript and approved its final version.

## Supplementary Material

Additional file 1 **Supplementary material**. This file contains supplementary text (describing data acquisition and applied methods) as well as supplementary table and figures. Click here for file
