# Supplementary material for: Phylogeny-guided interaction mapping in seven eukaryotes
Source: BMC Bioinformatics. 2009 Nov 30;10:393. doi: 10.1186/1471-2105-10-393 (PMC2793266; doi:10.1186/1471-2105-10-393)
Supplement: Additional file 1 — Supplementary material. This file contains supplementary text (describing data acquisition and applied methods) as well as supplementary table and figures. [file 1471-2105-10-393-S1.PDF]

Phylogeny-guided interaction mapping in seven eukaryotes (Supplementary material)

Janusz Dutkowski and Jerzy Tiuryn

{januszd,tiuryn}@mimuw.edu.pl

Institute of Informatics, University of Warsaw, Banacha 2, 02-097 Warsaw, Poland

Contents

1 Input datasets and data preprocessing **1**

2 Parameter settings **2**

3 Integrating datasets with different reliabilities and coverage **3**

4 Reference datasets **3**

5 Additional case studies **4**

6 Supplementary tables **6**

6.1 Table S1 - CAPPI-Pred and Interlog PPI predictions within selected functional modules 6

7 Supplementary figures **7**

7.1 Figure S1 - P-values and TP/FP ratios depending on the choice of threshold 7

7.2 Figure S2 - Inferred PPIs related to the *A. thaliana* SWI/SNF chromatin remodeling complex (extended view) 8

7.3 Figure S3 - Inferred PPIs within the human proteasome complex 9

7.4 Figure S4 - Inferred PPIs within the yeast endosome complex 10

1 Input datasets and data preprocessing

We have downloaded the protein sequence and annotation data from the Integr8 database [1] (December 2007 download). The input PPI data for the seven species was the same as in the InteroPorc implementation (interlog approach). The dataset included merged PPI data from the latest releases of three major databases: IntAct [2] (2008-08-22 version), MINT [3] (2008-05-16 version) and DIP

[4] (2008-07-08 version). We downloaded the input dataset (known interactions) for each species separately from the InteroPorc website `http://biodev.extra.cea.fr/interoporc/Default.aspx`.

Protein sequences were preprocessed leaving only the longest splice variants for each gene and clustered using the MCL algorithm [5], which identified 21759 disjoint protein families. We further filtered out the families which contained sequences from less than three species leaving 4083 conserved families. Additional 10 largest families were removed due to poor sequence overlap. For each of the remaining 4073 families we constructed a phylogenetic tree and reconciled it with the species tree of the seven organisms. For the purpose of small-scale case studies we made minor corrections in six families (adding proteins missed by the automated preprocessing of the Integr8 sequence database). All other steps of the analysis were performed automatically without manual curation.

2 Parameter settings

The downloaded PPI data was split by species and source experiments (according to PubMed ID). Reliability parameters for large-scale input dataset used in CAPPI-Integ and CAPPI-Pred were based on the estimates from [6], [7] and [8]:

First author	Year	Reliability
Gavin	2006	0.28
Giot	2003	0.2
Krogan	2006	0.27
Ewing	2007	0.7
Ito	2001	0.18
Li	2004	0.29
Gavin	2002	0.67
Ho	2002	0.27
Steltz	2005	0.15
Rual	2005	0.32
Hazbun	2003	0.31
Stanyon	2004	0.7
Formstecher	2005	0.45
Bouwmeester	2004	0.72
Uetz	2000	0.53

Smaller datasets were merged into one single dataset with reliability 0.9. During initial tests, the method showed that it was robust to variation in reliability parameters over a wide range of

values. Thus no special optimization was necessary. The number of true interactions in each species was estimated as in [9].

In case of CAPPI-Integ-3sp, in order to enable direct comparison with the Domain-ML approach, we set the false positive rate of each experiment to 0.0003 and the false negative rate of each experiment to 0.85, as was done by [10].

The parameters of the model of network evolution were set (in all cases) to the following conservative values: $p_d = 0.95$, $\delta_d = 0.001$, $p_s = 0.99$ and $\delta_s = 0.001$.

3 Integrating datasets with different reliabilities and coverage

The conditional probabilities corresponding to true positive rate, false positive rate, false negative rate, and true negative rate of each experiment were computed as follows:

$$Pr(X^{o_h^{(i)}}_{n_x,n_y} = 1 | X^{G_{i,m_i}}_{n_x,n_y} = 1) = \frac{Rel(o_h^{(i)})|o_h^{(i)}|}{|E_{i,m_i}|}$$

$$Pr(X^{o_h^{(i)}}_{n_x,n_y} = 1 | X^{G_{i,m_i}}_{n_x,n_y} = 0) = \frac{(1 - Rel(o_h^{(i)}))|o_h^{(i)}|}{|E'_{i,m_i}|}$$

$$Pr(X^{o_h^{(i)}}_{n_x,n_y} = 0 | X^{G_{i,m_i}}_{n_x,n_y} = 1) = 1 - \frac{Rel(o_h^{(i)})|o_h^{(i)}|}{|E_{i,m_i}|}$$

$$Pr(X^{o_h^{(i)}}_{n_x,n_y} = 0 | X^{G_{i,m_i}}_{n_x,n_y} = 0) = 1 - \frac{(1 - Rel(o_h^{(i)}))|o_h^{(i)}|}{|E'_{i,m_i}|}.$$

4 Reference datasets

The GO annotations for considered proteins and background protein populations were downloaded from the Integr8 database (December 2008 download). The functional similarity scores were computed separately for each protein pair using the SemSim Bioconductor package (`http://www.bioconductor.org/packages/2.0/bioc/html/SemSim.html`) and averaged over the number of predicted interactions or interactions in the input datasets.

Our second kind of quality assessment was based on estimating the ratio of true positive and false positive interactions. We used separate reference datasets to determine binary and co-complex true positive PPIs. Protein pairs which where not found in the reference dataset and had differential sub-cellular localizations were counted as false positives. Below we list the reference datasets used in each case.

Yeast reference datasets A set of 3388 gold-standard yeast binary PPIs was prepared by merging the LC-multiple set from [11] and gold standard dataset Binary-GS from [12], both downloaded from `http://interactome.dfci.harvard.edu/S_cerevisiae/host.php?page=download`.

The co-complex reference dataset of 21069 protein pairs was comprised by extracting pairs of proteins from yeast complexes listed in the MIPS complex catalog of [13] (`ftp://ftpmips.gsf.de/yeast/catalogues/complexcat/`) and in the CYC2008 Complex dataset of [14] (`http://wodaklab.org/cyc2008/downloads`). We discarded the complexes identified in high-throughput experiments (MIPS category 550). For CAPPI-Integ validation the complex and binary reference datasets were merged. For CAPPI-Pred experiments the binary and co-complex reference datasets were used separately and an additional dataset of experimental PPIs (binary and co-complex) was comprised from all previous reference datasets and from the left-out yeast input data, as well as a recent Y2H experiment CCSB-YI1 [12] (totaling 73252 PPIs altogether). Sub-cellular localization for yeast proteins were extracted from the MIPS sub-cellular catalog. Altogether there were 4857065 differentially localized protein pairs. All datasets were most recent as of December 2008.

Human reference datasets A reference set of 36244 binary PPIs was comprised from interactions downloaded from the HPRD database [15], which stores curated interactions, mostly from small-scale studies. Co-complex pairs were extracted from HPRD complexes (9669 pairs). For CAPPI-Pred experiments the binary and co-complex reference datasets were used separately and an additional dataset (All) of experimental PPIs (binary and co-complex) was comprised from all previous reference datasets and from the left-out human input data (totaling 57093 PPIs altogether). Sub-cellular localization for human proteins were extracted from the HPRD sub-cellular catalog. Altogether there were 41647579 differentially localized protein pairs. Most recent HPRD datasets were downloaded in August 2008.

5 Additional case studies

Human proteasome PPI predictions The result of our predictions of subunit-subunit interactions in the human proteasome is depicted in Figure S3. Compared to the yeast proteasome map, the human subnetwork contains more previously unreported interactions, possibly due to the incompleteness of the human data. Again, the resulting graph is split into four parts, each corresponding to a distinct subcomplex. The α- and β-rings clique representation is very similar to that of the yeast proteasome. The base subcomplex also has a very dense set of interactions. Some of these PPIs, namely PSMC2–PSMC6 (Rpt1–Rpt4), PSMC2–PSMC5 (Rpt1–Rpt6) and PSMC4–PSMC4 (Rpt3–Rpt3) have only recently been reported [16]. Like in the case of the yeast proteasome, we notice a central position of PSMD9 (p27) with respect to AAA-ATPase subunits PSMC1-PSMC6 (Rpt1-Rpt6), which has also been reported in [16]. Due to these confirmed interactions we decided to merge this protein into the base subnetwork. We also observe that PSMD8 (Rpn12) of the lid is predicted to densely interact with the base proteins. PSMD1 (Rpn2) and PSMD2 (Rpn1) each have five predicted interactions with other lid members and none with the

base. Therefore we decided to move them into the lid subnetwork. In fact PSMD1 is described as the largest non-ATPase subunit of the 19S regulator lid by the Entrez Gene database, somewhat differently from its Rpn2 homolog in yeast (usually attributed to the base subcomplex). PSMD2, like its yeast homolog Rpn1, has many predicted interactions with both subcomplexes (α and β) of the 20S proteasome, however in human they are not confirmed. We also notice a dense set of interactions between the six AAA-ATPase subunits (PSMC1-PSMC6) and the 20S catalytic core. Many of these interactions have not been previously reported in the literature and could be used as hypothesis in verifying experiments. Compared to our result for yeast, our representation of the lid subcomplex of the human 19S proteasome lacks PSMD13 (Rpn9), which we did not find in the initial Integr8 dataset. In human, we find an additional transiently associated protein PSMD5 (S5b), which binds to PSMC2.

Yeast ESCRT PPI predictions The yeast endosome PPIs, as inferred by our method, are depicted in Figure S4. We find that almost all predicted interactions (except for five self loops) are supported by experimental studies. Similarly as in the human network, all five complexes discussed in [17] can be naturally retrieved from the presented graph. Like in the human ESCRT complexes, the yeast homologs of CHMP1B (DID2) and of CHMP5 (VPS60) well fit (graph-theoretically) to ESCRT-3. This again confirms the observation stated in [18], where the authors call these two proteins 'proposed regulatory members' of ESCRT-3. Similarly to the human endosome network, the topology of the identified interaction network suggests that VPS23 (STP22) may play an important role mediating the interactions between complexes, although at the selected threshold we did not identify its interactions with the ESCRT-3 and Vps4 complexes (as we did in the human example).

6 Supplementary tables

6.1 Table S1 - CAPPI-Pred and Interlog PPI predictions within selected functional modules

Species	Module	Known Interactions	CAPPI-Pred		Interlog	
			Confirmed	Unconfirmed	Confirmed	Unconfirmed
Human	Proteasome	249	144	155	117	86
	Endosome	104	49	49	4	0
	Exosome	101	53	76	7	10
Yeast	Proteasome	328	177	66	56	6
	Endosome	72	22	5	4	0
Arabidopsis	SWI/SNF	14	13	83	0	10

Comparison of CAPPI-Pred and Interlog PPI predictions within functional modules featured in case studies. The number of know interactions within each module and the number of confirmed and unconfirmed interactions predicted by CAPPI-Pred and the Interlog approach (InteroPORC) are provided. The restrictive Interlog mapping approach identifies fewer unconfirmed predictions (potential false-positives) but the number of confirmed interactions in most cases (except perhaps the proteasome complexes) is too low to provide meaningful information about the module architecture.

7 Supplementary figures

7.1 Figure S1 - *P*-values and TP/FP ratios depending on the choice of threshold

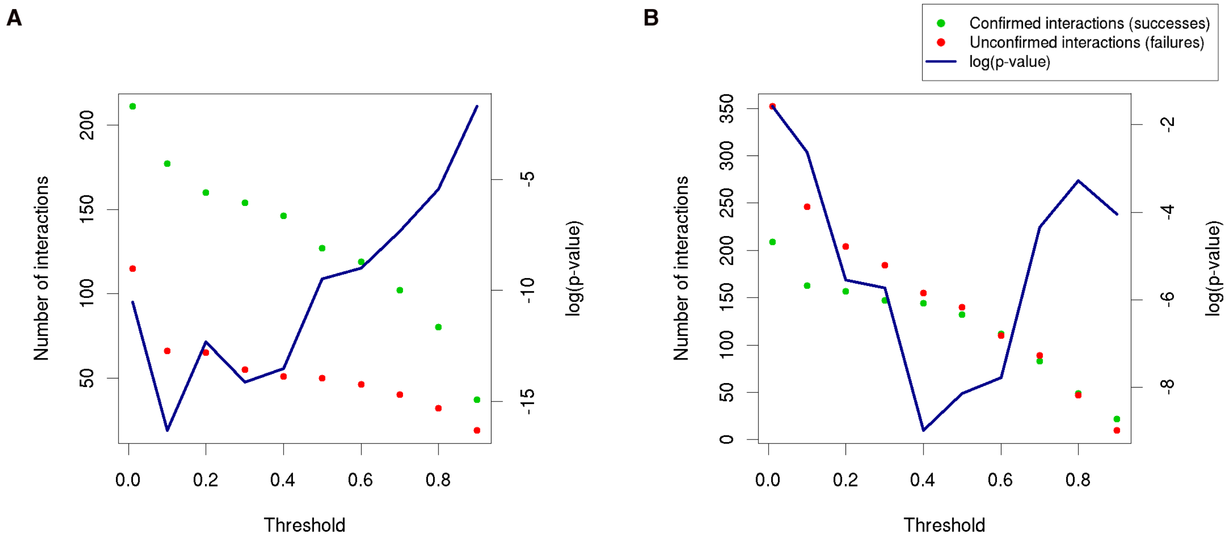

The number of confirmed predictions, unconfirmed predictions and corresponding p-values (in logarithmic scale), as a function of the threshold, for interactions among the 26S proteasome proteins from yeast (A) and human (B). The p-values are computed based on the hypergeometric distribution where confirmed interactions are considered as successes and unconfirmed interactions are considered as failures (Fisher's exact test). For both species CAPPI predictions are significant over a wide range of thresholds. The apparent threshold with the minimum p-value may serve as a point of reference at which predictions can be analyzed.

7.2 Figure S2 - Inferred PPIs related to the *A. thaliana* SWI/SNF chromatin remodeling complex (extended view)

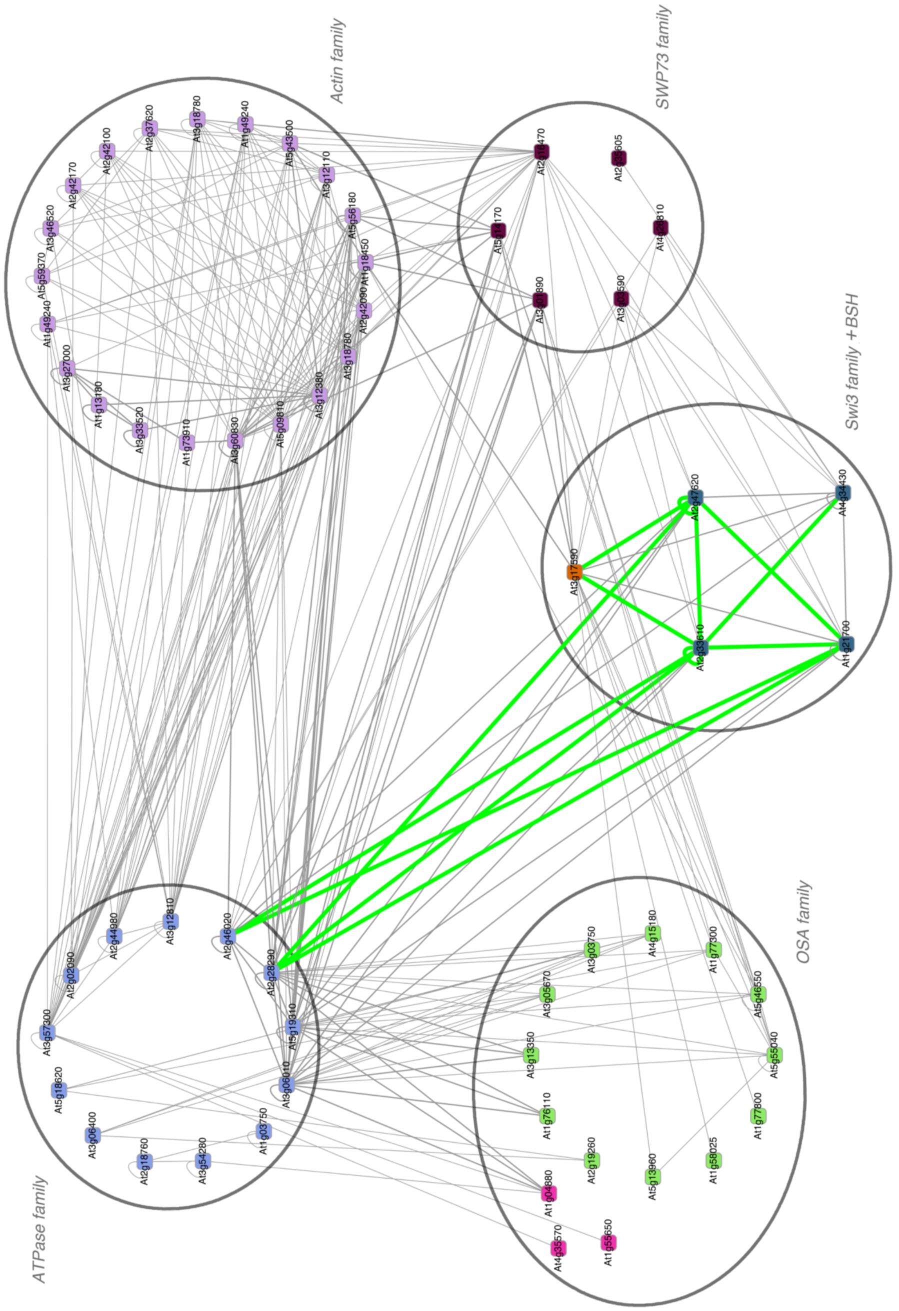

An extended view of the interaction network of the putative SWI/SNF complex in Arabidopsis as inferred by CAPPI-Pred. Nodes represent gene products and node colors represent protein families identified by sequence clustering. 13 of the predicted interactions which have been previously detected experimentally are denoted by green edges. 319 other PPI predictions are denoted by gray edges. The p-value of the predicted network is 3.411×10^{-9}.

7.3 Figure S3 - Inferred PPIs within the human proteasome complex

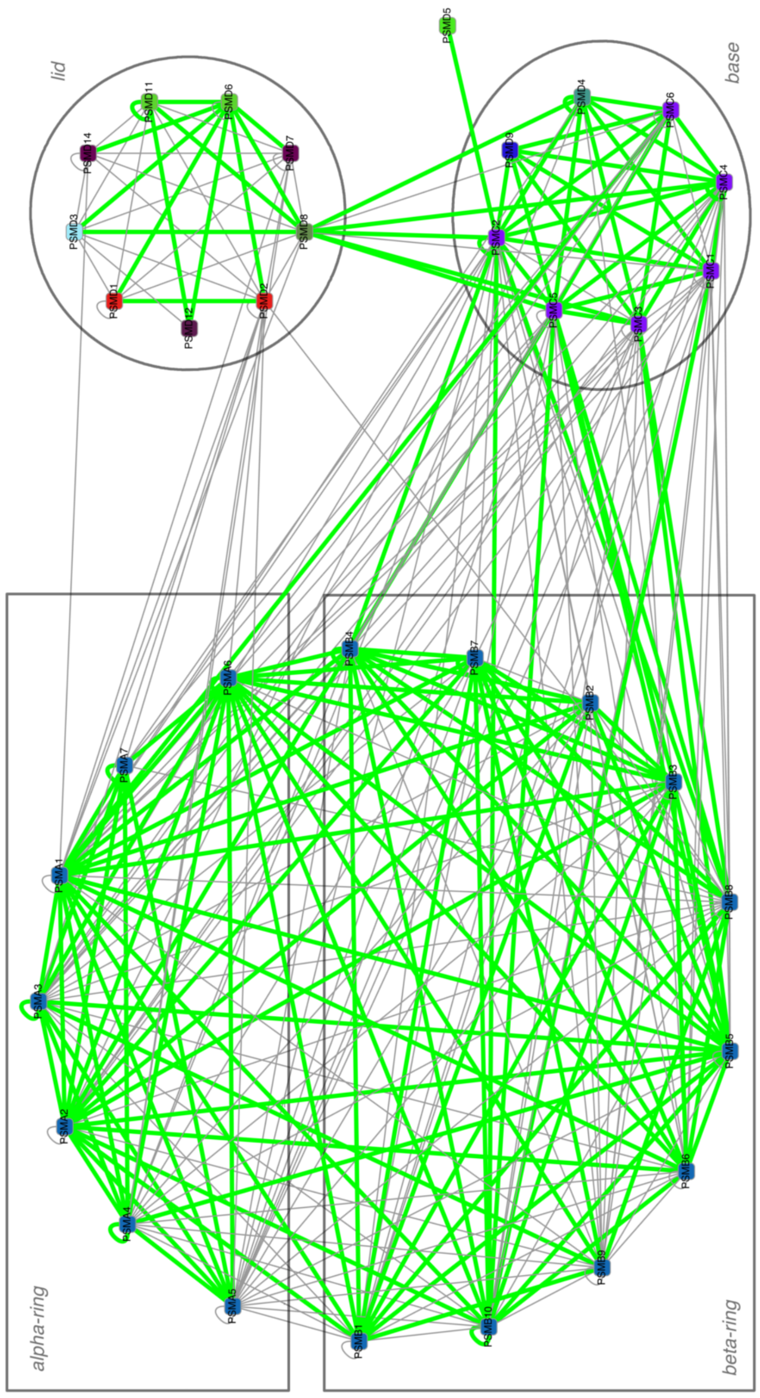

Interaction network of the human 26S proteasome complex as inferred by CAPPI-Pred. Nodes represent gene products and node colors represent protein families identified by sequence clustering. 144 of the predicted interactions which have been previously detected experimentally are denoted by green edges. 155 other PPI predictions are denoted by gray edges. The p-value of the predicted network is 1.614×10^{-6}.

7.4 Figure S4 - Inferred PPIs within the yeast endosome complex

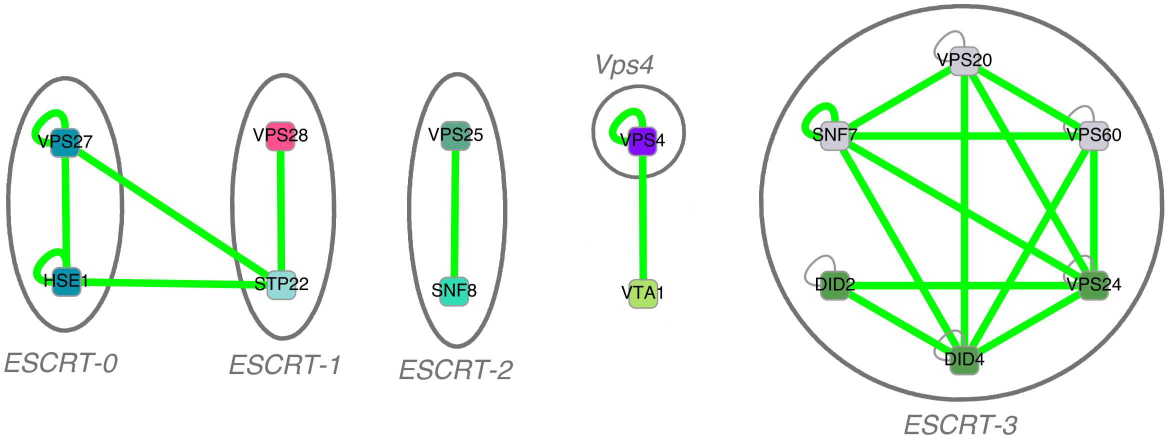

Interaction network of the yeast endosome complexes as inferred by CAPPI-Pred. Nodes represent gene products and node colors represent protein families identified by sequence clustering. 22 of the predicted interactions which have been previously detected experimentally are denoted by green edges. 5 other PPI predictions are denoted by gray edges. The p-value of the predicted network is 9.571×10^{-11}.

References

[1] Kersey P, Bower L, Morris L, Horne A, Petryszak R, Kanz C, Kanapin E, Das U, Michoud K, Phan I, Gattiker R, Kulikova T, Faruque N, Duggan K, Mclaren P, Reimholz B, Duret L, Penel S, Reuter I, Apweiler R: **Integr8 and Genome Reviews: integrated views of complete genomes and proteomes**. *Nucleic Acids Research* 2005, **33**:297–302.

[2] Hermjakob H, Montecchi-Palazzi L, Lewington C, Mudali S, Kerrien S, Orchard S, Vingron M, Roechert B, Roepstorff P, Valencia A, Margalit H, Armstrong J, Bairoch A, Cesareni G, Sherman D, Apweiler R: **IntAct: an open source molecular interaction database.** *Nucleic Acids Reseach* 2004, **32**(Database issue):D452–D455.

[3] Chatr-aryamontri A, Ceol A, Palazzi LM, Nardelli G, Schneider MV, Castagnoli L, Cesareni G: **MINT: the Molecular INTeraction database.** *Nucleic Acids Research* 2007, **35**(Database issue):D572–D574.

[4] Salwinski L, Miller CS, Smith AJ, Pettit FK, Bowie JU, Eisenberg D: **The Database of Interacting Proteins: 2004 update.** *Nucleic Acids Research* 2004, **32**(Database issue):D449–D451.

[5] Enright AJ, Van Dongen S, Ouzounis CA: **An efficient algorithm for large-scale detection of protein families.** *Nucleic Acids Research* 2002, **30**(7):1575–1584.

[6] Hart TG, Ramani AK, Marcotte EM: **How complete are current yeast and human protein-interaction networks?** *Genome Biology* 2006, **7**:120.

[7] Deng M, Sun F, Chen T: **Assessment of the reliability of protein-protein interactions and protein function prediction**. In *Proc. Eighth Pacific Symposium on Biocomputing* 2003:140–151.

[8] Patil A, Nakamura H: **Filtering high-throughput protein-protein interaction data using a combination of genomic features.** *BMC Bioinformatics* 2005, **6**:100.

[9] Stumpf MP, Thorne T, de Silva E, Stewart R, An HJ, Lappe M, Wiuf C: **Estimating the size of the human interactome**. *Proc Natl Acad Sci U S A* 2008, **105**:6959–6964.

[10] Liu Y, Liu N, Zhao H: **Inferring protein-protein interactions through high-throughput interaction data from diverse organisms.** *Bioinformatics* 2005, **21**:3279–3285.

[11] Reguly T, Breitkreutz A, Boucher L, Breitkreutz BJ, Hon G, Myers C, Parsons A, Friesen H, Oughtred R, Tong A, Stark C, Ho Y, Botstein D, Andrews B, Boone C, Troyanskya O, Ideker T, Dolinski K, Batada N, Tyers M: **Comprehensive curation and analysis of global interaction networks in Saccharomyces cerevisiae.** *Journal of Biology* 2006, **5**(11).

[12] Yu H, Braun P, Yildirim MA, Lemmens I, Venkatesan K, Sahalie J, Hirozane-Kishikawa T, Gebreab F, Li N, Simonis N, Hao T, Rual JF, Dricot A, Vazquez A, Murray RR, Simon C, Tardivo L, Tam S, Svrzikapa N, Fan C, de Smet AS, Motyl A, Hudson ME, Park J, Xin X, Cusick ME, Moore T, Boone C, Snyder M, Roth FP, Barabasi AL, Tavernier J, Hill DE, Vidal M: **High-Quality Binary Protein Interaction Map of the Yeast Interactome Network**. *Science* 2008, **3**:104–110.

[13] Mewes HW, Frishman D, Mayer KF, Münsterkötter M, Noubibou O, Pagel P, Rattei T, Oesterheld M, Ruepp A, Sümpflen V: **MIPS: analysis and annotation of proteins from whole genomes in 2005.** *Nucleic Acids Research* 2006, **34**(Database issue):D169–D172.

[14] Pu S, Wong J, Turner B, Cho E, Wodak SJ: **Up-to-date catalogues of yeast protein complexes**. *Nucleic Acids Research* 2009, **37**:825–831.

[15] Prasad KTS, Goel R, Kandasamy K, Keerthikumar S, Kumar S, Mathivanan S, Telikicherla D, Raju R, Shafreen B, Venugopal A, Balakrishnan L, Marimuthu A, Banerjee S, Somanathan DS, Sebastian A, Rani S, Ray S, Kishore HCJ, Kanth S, Ahmed M, Kashyap MK, Mohmood R, Ramachandra YL, Krishna V, Rahiman AB, Mohan S, Ranganathan P, Ramabadran S, Chaerkady R, Pandey A: **Human Protein Reference Database – 2009 update**. *Nucleic Acids Research* 2008, **37**:D767–D772.

[16] Chen C, Huang C, Chen S, Liang J, Lin W, Ke G, Zhang H, Wang B, Huang J, Han Z, Ma L, Huo K, Yang X, Yang P, He F, Tao T: **Subunit-subunit interactions in the human 26S proteasome.** *Proteomics* 2008, **8**:508–520.

[17] Hurley JH, Emr SD: **The ESCRT complexes: structure and mechanism of a membrane-trafficking network.** *Annual Review of Biophysics and Biomolecular Structure* 2006, **35**:277–298.

[18] Shim S, Merrill SA, Hanson PI: **Novel interactions of ESCRT-III with LIP5 and VPS4 and their implications for ESCRT-III disassembly.** *Molecular Biology of the Cell* 2008, **19**:2661–2672.
